# Dapsone-induced methemoglobinemia in a child with leprosy^☆^

**DOI:** 10.1016/j.abd.2024.05.006

**Published:** 2025-01-09

**Authors:** John Verrinder Veasey, Bruna Cavaleiro de Macedo Souza, Guilherme Camargo Julio Valinoto

**Affiliations:** a Dermatology Clinic, Hospital da Santa Casa de São Paulo, São Paulo, SP, Brazil; b Discipline of Dermatology, Faculty of Medical Sciences, Santa Casa de São Paulo, São Paulo, SP, Brazil

Dear Editor,

Methemoglobinemia may result from inherited or acquired processes.^1^, ^2^ The acquired forms are the most common, mainly due to exposure to substances that directly or indirectly cause hemoglobin oxidation, such as dapsone.^1^ The diagnosis should be suspected in cases of unexplained cyanosis and hypoxemia.^2^ The present report describes a patient diagnosed with multibacillary leprosy who developed methemoglobinemia due to the use of polychemotherapy to treat her infection.

An 11-year-old female patient, weighing 31 kg, from the state of Maranhão and living in the city of São Paulo for three years, had a family history of an uncle and cousin treated for leprosy in her state of origin. She reported changes in finger sensitivity and a burning sensation on the left hand, associated with the appearance of disseminated hypochromic macules on the trunk (Fig. 1). A histamine test was performed on one of the lesions on the back, which showed an incomplete response with absence of the second stage of the Lewis triple reaction (Fig. 2), and histopathology of skin biopsy showed a superficial and deep histiocytic dermatitis with neural aggression and the presence of acid-fast bacilli on staining the Ziehl-Neelsen method, thus concluding the diagnosis of multibacillary leprosy. Laboratory tests including G6PD were performed and showed no relevant alterations, and treatment with pediatric polychemotherapy (PCT) was initiated (supervised monthly dose: rifampicin 450 mg, clofazimine 150 mg and dapsone 50 mg; daily self-administered dose: clofazimine 50 mg and clofazimine 50 mg) for the treatment of multibacillary leprosy.

**Fig. 1:**
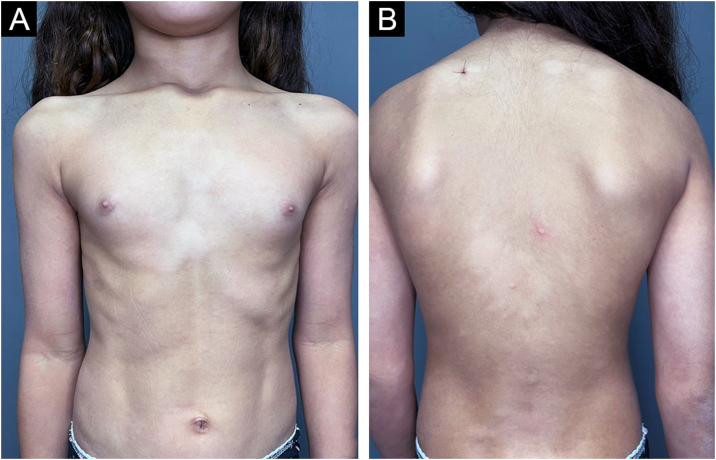
Clinical aspect of the diagnosis of leprosy, with multiple hypochromic macules distributed on the anterior and posterior trunk.

**Fig. 2:**
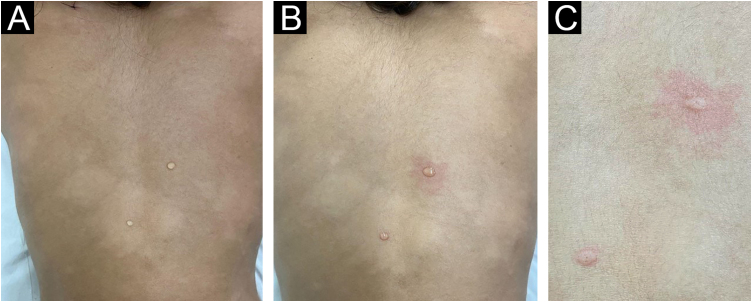
Histamine test in a patient with leprosy. (A) A drop of histamine is applied to a hypochromic lesion and to healthy skin, followed by histamine punctures. (B) The reflex erythema present in healthy skin is not evident in the leprosy lesion. (C) Detail of erythematous papules in both punctures but with reflex erythema only in healthy skin.

As soon as she finished the 3rd blister pack of PCT, the patient presented dyspnea, fatigue and central cyanosis (Fig. 3), needing medical help. An immediate investigation was carried out, and the patient showed peripheral saturation in ambient air of 88%, with O_2_ saturation in arterial blood gas of 100% and a serum methemoglobin level of 3.8% (reference: < 2%). With the confirmed diagnosis of mild methemoglobinemia secondary to dapsone in the PCT, the patient remained under observation with oxygenation via catheter and hydration, which reversed the condition within 24 hours. Dapsone was replaced by minocycline, and the patient continues to be regularly monitored at the health service, having finished nine blister packs to date.

**Fig. 3:**
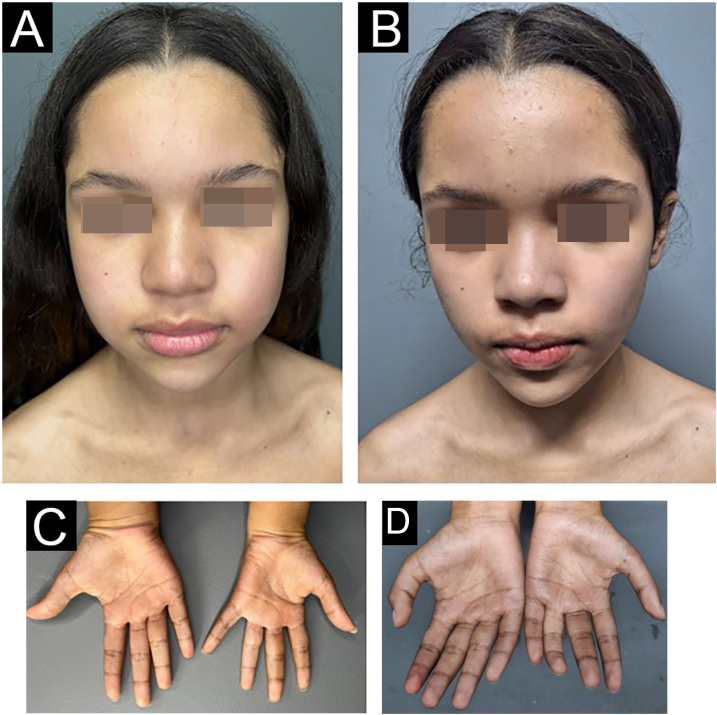
Patient before treatment (A and C) and on the day of the diagnosis of methemoglobinemia (B and D) showing central and extremity cyanosis, associated with complaints of dyspnea and fatigue.

The occurrence of cases of leprosy in children under 15 years of age indicates foci of active transmission, an important indicator for monitoring endemic disease.^3^, ^4^ From 2012 to 2021, 17,442 new cases of leprosy were diagnosed in children under 15 years of age in Brazil, with a 64% reduction in the detection rate of new cases during the period.^5^ Despite variations in clinical forms, 2020 data from the Brazilian Ministry of Health show that 78.2% of pediatric cases are classified as multibacillary, increasing the propensity for reactions, complications, and sequelae.^5^

The diagnosis of leprosy is based on the clinical aspect and is complemented by propaedeutic methods of sensitivity assessment, bacilloscopy, and histopathology of the skin.^3^, ^4^ The use of the histamine test is highly relevant in pediatric cases, where the patient may not adequately understand the neurosensory propaedeutic analysis, resulting in inconclusive tests.^6^ The absence of reflex erythema in the lesion suspected of leprosy demonstrates damage to nerve endings involved in cutaneous vasodilation, evidencing the damage to the fibers caused the bacillus. In addition to providing proof of clinical suspicion, it has very low morbidity when compared to other procedures, such as bacilloscopy and skin biopsy.^6^ The case described herein clearly illustrates the advantages of this test.

The first-line treatment of multibacillary leprosy is carried out with PCT consisting of rifampicin, dapsone, and clofazimine over 12 months.^7^ It requires close monitoring to adjust doses and identify potential side effects, including methemoglobinemia resulting from the use of dapsone, which is the drug most frequently associated with this complication.^1^, ^8^

Methemoglobinemia is a rare disease associated with the oxidation of the ferrous ion (Fe^2+^) of hemoglobin to ferric ion (Fe^3+^), converting it into methemoglobin, which is more avid for oxygen and incapable of transferring it to tissues.^2^ The severity of the disease depends on the percentage of methemoglobin (MetHb), the rate of increase in MetHb levels, the patient intrinsic ability to eliminate it, and the patient underlying functional status.^2^, ^8^ Clinical suspicion arises in patients with cyanosis and hypoxemia of uncertain etiology associated with a difference in saturation between oxygen measured by pulse oximetry and arterial blood gas analysis.^1^, ^2^, ^7^ The case described herein clearly illustrates this condition, confirmed by a methemoglobin level slightly above the reference value. The control of mild cases is achieved with supportive care and discontinuation of the related medication, while methylene blue and vitamin C are indicated for moderate to severe forms.^8^, ^9^, ^10^

The use of minocycline or ofloxacin is indicated as replacement therapy in patients who react to dapsone in PCT.^7^ Because the present patient was a pediatric one, minocycline was chosen, since quinolones are related to the early closure of metaphyseal plates, prematurely interrupting patient growth.^6^

## Financial support

None declared.

## Authors' contributions

Bruna Cavaleiro de Macedo Souza: Design and planning of the study; data collection, or analysis and interpretation of data; statistical analysis; drafting and editing of the manuscript or critical review of important intellectual content; collection, analysis and interpretation of data; effective participation in research orientation; intellectual participation in the propaedeutic and/or therapeutic conduct of the studied cases; critical review of the literature; approval of the final version of the manuscript.

John Verrinder Veasey: Design and planning of the study; data collection, or analysis and interpretation of data; statistical analysis; drafting and editing of the manuscript or critical review of important intellectual content; collection, analysis and interpretation of data; effective participation in research orientation; intellectual participation in the propaedeutic and/or therapeutic conduct of the studied cases; critical review of the literature; approval of the final version of the manuscript.

Guilherme Camargo Julio Valinoto: Design and planning of the study; data collection, or analysis and interpretation of data; statistical analysis; drafting and editing of the manuscript or critical review of important intellectual content; collection, analysis and interpretation of data; effective participation in research orientation; intellectual participation in the propaedeutic and/or therapeutic conduct of the studied cases; critical review of the literature; approval of the final version of the manuscript.

## Conflicts of interest

None declared.
